# A photographic data set of reef and coral communities across Venezuela

**DOI:** 10.1016/j.dib.2021.107235

**Published:** 2021-06-17

**Authors:** Luis M. Montilla, Emy Miyazawa, Alejandra Verde, Esteban Agudo-Adriani, Alfredo Ascanio, José Cappelletto, María López-Hernández, Gloria Mariño-Briceño, Stephanie Martínez, Zlatka Rebolledo-Sánchez, Andreína Rivera, Daniela S. Mancilla, Aldo Cróquer

**Affiliations:** a Laboratorio de Ecología Experimental, Simón Bolívar University, Venezuela; b Grupo de Investigación y Desarrollo en Mecatrónica, Simón Bolívar University, Venezuela

**Keywords:** Coral reefs, Venezuela, Caribbean, Benthic communities, Phototransects

## Abstract

This dataset contains 2850 photographs of the seafloor in coral communities from Venezuela that were taken during 2017 and 2018. We used a hierarchical experimental design with four random factors representing four different spatial scales: (1) region (hundreds of kilometers), (2) localities (tens of kilometers), (2) reef sites (hundreds of meters) and (3) transects (a couple meters) across the Venezuelan coast. At each site, four 30-m transects were deployed parallel to the coastline, and 15 pictures were taken every other meter at each transect, containing an area of at least 80 × 90cm with enough resolution to identify benthic groups. This dataset covers spatial scales from a few meters to hundreds of kilometers; marine protected areas, and non-protected areas; coastal zones, continental and oceanic islands. These images have the potential to be further used for training researchers in benthic organisms identification, and training artificial intelligence classification algorithms. Also, they represent and updated baseline to perform spatial and temporal comparisons in Venezuela or further studies involving multiple spatial scales in the region.

## Specifications Table

**Table:** 

Subject	Ecology
Specific subject area	Coral Reef Ecology
Type of data	JPG Images
How data were acquired	Images were taken using a Nikon Coolpix AW130 or a SeaLife Micro 2.0
Data format	Raw
Parameters for data collection	Selected sites had to have shallow coralline communities (between 8 and 10m) with enough visibility to photograph the bottom and be able to identify the organisms at the best taxonomic resolution possible. Each Locality had to have at least three different sites, separated by several hundred meters.
Description of data collection	All surveys were conducted using scuba equipment between 8-10 meters depth. At each site, four 30-m transects were deployed at least five meters apart, and 15 pictures were taken every other meter at each transect. One of four types of frames were included in the picture for scaling: (1) a yellow-black bar indicating 10cm for each coloured segment; (2) an L-shaped bar with perforations, the separation between two perforations being 5cm; (3) a regular bar with perforations every 10 cm. (4) a regular bar with yellow tape separated by 10 cm from the external borders of the tape.
Data source location	Venezuela. For details, please see Table 1.
Data accessibility	Repository name: OSF Data identification number: DOI https://doi.org/10.17605/OSF.IO/HE6QW Direct URL to data: https://osf.io/he6qw/

## Value of the Data

• This photographic dataset represents the first publicly available repository of images from coral habitats in Venezuela across different spatial scales (from a few meters to hundreds of kilometres), encompassing marine protected areas, and non-protected areas; coastal zones and continental and oceanic islands. The data set can be used to determine the structure of benthic communities and associated with coral reefs and coralline habitats. These data can allow to further insights about the benthic community structure associated with coral reefs and coralline communities from late 2017 to mid 2018 in the Southern Caribbean. The data represents a baseline for this period that will be valuable for purposes of comparisons in times of rapid climate change.
• These data are available for all the scientific community, students, conservation practitioners and marine protected areas policymakers and managers.
• These images can be used for capacity building through the identification of species and/or major benthic taxa, and to train and test artificial intelligence models. They also represent a baseline of the surveyed communities for future spatial and temporal comparisons or regional studies encompassing the Caribbean basin.

## 1 Data Description

Here we provide a collection of 2850 photographs as jpeg files. They contain an area of at least 80 × 90cm (with the only exception of the locality Ocumare, where the area of the quadrat is variable in the even transects) of the seafloor in coral communities in Venezuela during 2017 and 2018. This dataset includes 36 sites encompassing the Venezuelan coast, with enough resolution to identify benthic groups and up to taxonomic species in many cases. These images can be analysed with software like PhotoQuad [1], CPCe [2], or CoralNet [3] to generate multivariate data of benthic communities at different taxonomic and functional resolutions. These images were used in the research articles Miyazawa et al. [4] and Montilla et al. [5]. The locations are listed in Table 1:

**Table 1:** List of sites included in the present photographic record.

Locality	Site	Country	Transects	Latitude	Longitude	Year	Month	MPA
Morrocoy	Mero	Venezuela	4	10.81969444	-68.24771667	2017	Mar	1
Morrocoy	Sur	Venezuela	4	10.72943611	-68.24435556	2017	Mar	1
Morrocoy	Bajo Caiman	Venezuela	4	10.849223	-68.236805	2017	Mar	1
Morrocoy	Boca Seca	Venezuela	4	10.83360556	-68.23656111	2017	Mar	1
Morrocoy	Medio	Venezuela	4	10.74233889	-68.23593889	2017	Mar	1
Morrocoy	Sombrero	Venezuela	4	10.88862222	-68.21460833	2017	Mar	1
Morrocoy	Norte	Venezuela	4	10.77952222	-68.200675	2017	Mar	1
Ocumare	Guabinitas	Venezuela	4	10.48401667	-67.82534167	2018	Feb	0
Ocumare	Cienaga oeste	Venezuela	4	10.47823889	-67.80893333	2018	Feb	0
Ocumare	Cienaga interno	Venezuela	4	10.47502778	-67.80861111	2018	Feb	0
Ocumare	Cienaga este	Venezuela	4	10.48655556	-67.80427778	2018	Feb	0
Chichiriviche	Petaquire	Venezuela	3	10.54826667	-67.27703333	2017	Oct	0
Chichiriviche	Playa Tiburon	Venezuela	4	10.54741944	-67.26705	2017	Oct	0
Chichiriviche	Punta Mono	Venezuela	4	10.55093611	-67.25188333	2017	Oct	0
Chichiriviche	La Pared	Venezuela	4	10.55367778	-67.23888333	2018	Jan	0
Chichiriviche	Media Legua	Venezuela	4	10.55511389	-67.21023333	2017	Oct	0
Chichiriviche	Punta de Media Legua	Venezuela	4	10.55735	-67.20318889	2017	Oct	0
Los Roques	Cayo de Agua	Venezuela	4	11.81727778	-66.94033333	2017	Jan	1
Los Roques	Dos Mosquises	Venezuela	4	11.79266667	-66.89563889	2017	Jan	1
Los Roques	Las Salinas	Venezuela	4	11.74016667	-66.80748889	2017	Jan	1
Los Roques	La Venada	Venezuela	4	11.87865	-66.72434167	2017	Jan	1
Los Roques	Cote	Venezuela	4	11.77330556	-66.72144444	2017	Jan	1
Los Roques	Rabuski	Venezuela	4	11.882225	-66.68893889	2017	Jan	1
Los Roques	Madriski	Venezuela	4	11.93988611	-66.66118889	2017	Jan	1
Mochima	Carabela	Venezuela	4	10.38655556	-64.37455556	2017	Apr	1
Mochima	Punta Cruz	Venezuela	4	10.39555556	-64.36752778	2017	Apr	1
Mochima	San Agustin	Venezuela	4	10.38188889	-64.34730556	2017	Apr	1
Mochima	Garrapata	Venezuela	4	10.38066667	-64.33766667	2017	Apr	1
Mochima	Gabarra	Venezuela	4	10.38205	-64.33766	2017	Apr	1
Mochima	Blanca	Venezuela	4	10.39402778	-64.33480556	2017	Apr	1
Cubagua	Charagato	Venezuela	4	10.81556389	-64.22708889	2017	Oct	0
Cubagua	La Muerta	Venezuela	4	10.79978889	-64.217275	2017	Oct	0
Cubagua	Punta Conejo	Venezuela	4	10.83033056	-64.16479444	2017	Oct	0
Los Frailes	Cominoto	Venezuela	4	11.21461944	-63.76818056	2017	Oct	0
Los Frailes	La Pecha	Venezuela	4	11.20229167	-63.75253889	2017	Oct	0
Los Frailes	Puerto Real	Venezuela	4	11.18772778	-63.72993056	2017	Oct	0

## 2 Experimental Design, Materials and Methods

### 2.1 Experimental design

The surveys were designed to test hypotheses regarding coral communities variation at several spatial scales. We considered in our experimental design four factors:

• Region: random factor, with three levels: Eastern, Central, and Western regions of the Venezuelan coast.
• Locality: random factor and nested to Region, with a total of seven levels, distributed as two eastern locations, two central locations and three western locations.
• Site: random factor, nested to Locality, with a total of 36 levels. Each locality included three to seven sites.
• Transect: random factor, nested to Site, with a total of 143 levels, four transects for each site.

### 2.2 Field methods

At each selected site, a team of divers deployed four 30m-long random transects at a minimum depth of 8 meters and a maximum of 10m. Each transect had a separation of at least 5m. 15 photographs of the bottom were taken every other meter. One of four types of frames were included in the picture for scaling: (1) a yellow-black bar indicating 10cm for each coloured segment; (2) an L-shaped bar with perforations, the separation between two perforations being 5cm; (3) a regular bar with perforations every 10 cm. (4) a regular bar with yellow tape separated by 10 cm from the external borders of the tape.

## Ethics Statement

Authors did not use any animal or human experimental materials, and are not, therefore, subject to their ethical concerns

## CRediT Author Statement

**Luis M. Montilla:** Formal analysis, Investigation, Data Curation, Writing – original draft; **Emy Miyazawa:** Formal analysis, Investigation, Data Curation, Writing – review & editing; **Alejandra Verde:** Investigation, Writing – review & editing; **Esteban Agudo-Adriani:** Investigation, Writing – review & editing; **Alfredo Ascanio:** Investigation, Writing – review & editing; **José Cappelletto:** Software, Investigation, Writing – review & editing; **María López-Hernández:** Investigation, Writing – review & editing; **Gloria Mariño-Briceño:** Investigation, Writing – review & editing; **Stephanie Martínez:** Investigation, Writing – review & editing; **Zlatka Rebolledo-Sánchez:** Investigation, Writing – review & editing; **Andreína Rivera:** Investigation, Writing – review & editing; **Daniela S. Mancilla:** Investigation, Writing – review & editing; **Aldo Cróquer:** Conceptualization, Methodology, Investigation, Resources, Writing – review & editing, Supervision, Project administration, Funding acquisition.

## Declaration of Competing Interest

This research was funded by Waitt Foundation through the Rapid Ocean Conservation (ROC) Grant Program.

## References

[1] Trygonis V., Sini M. (2012). photoQuad: a dedicated seabed image processing software, and a comparative error analysis of four photoquadrat methods. J. Exp. Mar. Biol. Ecol..

[2] Kohler K.E., Gill S.M. (2006). Coral point count with excel extensions (CPCe): a visual basic program for the determination of coral and substrate coverage using random point count methodology. Comput. Geosci..

[3] Beijbom O., Edmunds P.J., Roelfsema C., Smith J., Kline D.I., Neal B.P., Dunlap M.J., Moriarty V., Fan T.-Y., Tan C.-J., Chan S., Treibitz T., Gamst A., Mitchell B.G., Kriegman D. (2015). Towards automated annotation of benthic survey images: variability of human experts and operational modes of automation. PLOS ONE.

[4] Miyazawa E., Montilla L.M., Agudo-Adriani E.A., Ascanio A., Mariño-Briceño G., Croquer A. (2020). On the importance of spatial scales on beta diversity of coral assemblages: a study from venezuelan coral reefs. Peer J..

[5] Montilla L.M., Miyazawa E., Ascanio A., López-Hernández M., Mariño-Briceño G., Rebolledo-Sánchez Z., Rivera A., Mancilla D.S., Verde A., Cróquer A. (2020). The use of pseudo-multivariate standard error to improve the sampling design of coral monitoring programs. Peer J..

